# Polyetherketoneketone (PEKK): An emerging biomaterial for oral implants and dental prostheses

**DOI:** 10.1016/j.jare.2020.09.004

**Published:** 2020-09-18

**Authors:** Hatim Alqurashi, Zohaib Khurshid, Azeem Ul Yaqin Syed, Syed Rashid Habib, Dinesh Rokaya, Muhammad Sohail Zafar

**Affiliations:** a School of Clinical Dentistry, University of Sheffield, United Kingdom; b Department of Preventive Dental Sciences, School of Dentistry, King Faisal University, Al-Ahsa 31982, Saudi Arabia; c Department of Prosthodontics and Dental Implantology, College of Dentistry, King Faisal University, Al-Ahsa 31982, Saudi Arabia; d Department of Prosthetic Dental Sciences, College of Dentistry, King Saud University, Riyadh 11545, Saudi Arabia; e Department of Clinical Dentistry, Walailak University International College of Dentistry, Bangkok 10400, Thailand; f Department of Restorative Dentistry, College of Dentistry, Taibah University, Madinah Al Munawwarah, Saudi Arabia; g Department of Dental Materials, Islamic International Dental College, Riphah International University, Islamabad 44000, Pakistan

**Keywords:** High-performance polymer, PEKK, Dental implants, Restorations, dental, Prosthodontics

## Abstract

Polyetherketoneketone (PEKK) is a new evolving polymeric material. The present article comprehensively reviewed an overview of various applications of PEKK in prosthodontics and oral implantology, highlighting its prospects for clinical applications. PEKK biomaterials is an elastic material with good shock absorbance and fracture resistance and present ultra-high performance among all thermoplastic composites for excellent mechanical strength, chemical resistance, and high thermal stability. Available articles on PEKK for dental applications were reviewed from January 1957 to August 2020) using MEDLINE/PubMed, Web of Science, and ScienceDirect resources. PEKK presents suitable physical, mechanical, and chemical properties for applications in prosthodontics and oral implantology. PEKK has good potential for a wide range of dental applications, including tooth restorations, crowns, bridge, endoposts, denture framework, implant-supported fixed prosthesis, and dental implants. PEKK dental implants have shown lesser stress shielding compared to titanium for dental implant applications. Further modifications and improving material properties can result in broader applications in the field of dentistry. Long term evaluations are needed as PEKK is recently applied in dentistry, and there are limited studies published on PEKK.

## Graphical abstract

**Figure:**
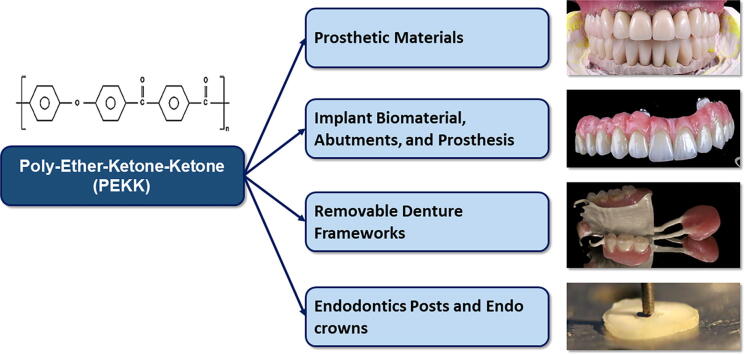

## Introduction

Polymers being one of the essential materials in dentistry, poses excellent physical, mechanical properties and are reported to have excellent biocompatibility. Various removable appliances, restorations, and denture base materials are fabricated from polymers [1], [2]. Polyetherketoneketone (PEKK) is a new polymeric material that has attracted the attention of researchers because of its excellent properties that can be used in many applications [3]. The PEKK is a methacrylate-free thermoplastic high-performance material [4]. PEKK was firstly introduced by Bonner in 1962 [5], and since then, it has been used for different industrial and military purposes [6]. Recently, PEKK has increasingly used as a biomaterial with properties suitable for dental and medical applications [7]. The PEKK has a wide range of applications in restorative, prosthetic, and implant dentistry. The PEKK is a promising material in the field of cranial and orthopedic implants. Their wide biomedical applications are because of its higher mechanical strength and the presence of the second ketone group that allows for more surface modification of its surface.

**Fig. 1:**
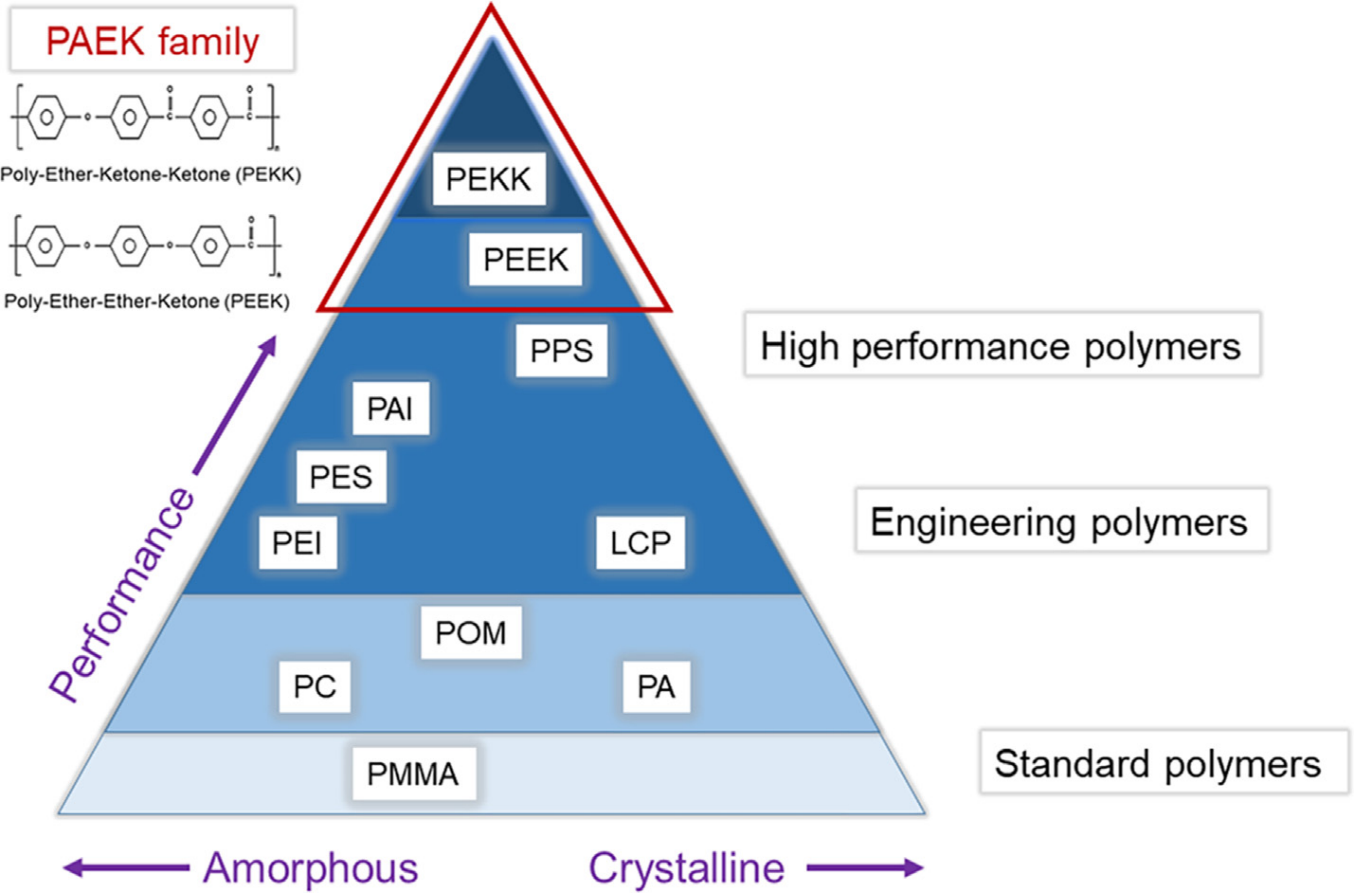
Structure and performance of PAEK (PEKK and PEEK), and fabrication of PEKK. (A) Performance of PAEK; (B) Structures of PAEK; (C) Production of PEKK by electrophilic substitution using nitrobenzene and aluminum chloride (AlCl_3_). PAEK = Polyaryletherketone, PEEK = Polyetheretherketone, PEKK = Polyetheretherketone, PPS = Polyphenylene sulfide, PAI = Polyamideimide, PES = Polyethersulfone, PEI = Polyethylenimine, POM = Polyoxymethylene, PC = Polycarbonates, PA = polyamide, PMMA = Polymethyl methacrylate.

The PEKK and polyetheretherketone (PEEK) are the two most well-known of the polyaryletherketone (PAEK) family. The PAEK family are thermoplastic polymers and have been in the engineering field since the 1980s and shows excellent mechanical properties and chemical resistance [8]. PAEK family show ultra-high performance (superior mechanical performances with chemical resistant) among all thermoplastic composites linked to their processing parameters (Fig. 1A) [4]. The PEEK emerged in the late 1990s as a semi-crystalline material and showed excellent biological, mechanical, and physical properties for biomedical applications [9], [10]. Promising applications of PEEK biomaterial are dental implant [3], temporary abutment and fixed prosthesis [11] and removable denture [12], and finger prosthesis [13]. These incredible outcomes of PEEK as dental materials attracted the attention of researchers to study the other members of the PAEK family, PEKK. Available articles on PEKK for dental applications were reviewed from January 1957 to August 2020) were reviewed using MEDLINE/PubMed, Web of Science, and ScienceDirect resources. This article presents an overview of PEKK and its various applications in restorative, prosthetic, and implant dentistry.

## Structure and synthesis of PEKK

The PAEK is a linear aromatic polyether ketone represented by ultrahigh molecular weight polyethylene. The structure of PEEK and PEKK have aromatic rings, which differ in the ratio of ether- and keto- group (Fig. 1B) [11]. There are some differences between PEKK and PEEK. PEKK has a second ketone group, and it increases polarity and backbone rigidity, which results in an increase in glass transition and melting temperature [14]. Moreover, PEKK displays both amorphous and crystalline behavior, and different products can be produced. A PEKK with 60% straight and 40% kinked segments melts at 305 °C but PEEK with 80% straight and 20% kinked melts at 360 °C. In addition, the extra ketone group in PEKK has strong polymer chains and shows better physical and mechanical properties, such as compressive strength [15].

PEKK is a liner thermoplastic polymer and consists of a benzene ring attached consecutively by ether or ketone- groups (Fig. 1B) [10]. PEKK can be produced from diphenyl ether and iso- and terephthaloyl chlorides with aluminum chloride (AlCl_3_) and nitrobenzene (Fig. 1C) [14].

## Properties of PEKK

### Physical and mechanical properties

PEKK shows excellent physical and mechanical properties, such as melting temperature and compressive strength in comparison to other polymeric materials [15]. In comparison to PEEK (pure and glass-reinforced), PEKK shows better mechanical properties in terms of flexure, tensile, and compressive strength [16]. Pekkton® ivory (Cendres + Métaux, SA, Switzerland), a product of PEKK has 80% higher compressive strength compared to un-reinforced PEEK [17]. The addition of titanium dioxide (TiO_2_) in PEKK, increases the hardness and wear resistance [18].

The shock absorbance with suitable strength (65 MPa) and fracture resistance properties of PEKK raises the possibility of using it as restorative material [19], [20]. The PEKK has similar compression strength with a lesser modulus of elasticity compared to dentin [20]. Similar to PEEK, the elastic modulus of PEKK is comparable to those of bone. Hence, PEKK can be used as a dental implant biomaterial for excellent mechanical properties and better stress distribution (Table 1, Table 2). Recently, Alsadon et al. evaluated the fatigue behavior of PEKK bilayered crowns in comparison to zirconia and nickel chromium-based crowns [16]. The fatigue limit of PEKK (754 N) was reported remarkably higher compared to zirconia (422 N), and nickel-chromium (586 N). Similarly, the fatigue limit of PEKK composite veneered molar crowns is also comparable with the cobalt-chromium and polymethylmethacrylate (PMMA) (750 N). According to Burke’s classification, the fracture code of PEKK was distributed between code one and two while Zr and NiCr exhibited code one and distribution between code 1 and 4, respectively, when subjected to loading below the group’s fatigue limit [16].

**Table 1:** Comparison of mechanical properties between PEKK and some human structures.

Materials	Tensile strength (MPa)	Elastic modulus (GPa)	Flexural strength (MPa)	Reference
PEKK	115	5.1	140–200	[15]
Cortical bone	104–121	14	50–150	[3], [21]
Cancellous bone	10–20	1.37	10–20	[3], [21]
Dentine	104	15	212.9	[3], [22]
Enamel	67.5	40–83	NA	[3]
Titanium	954	102–110	65	[3], [23]

**Table 2:** Mechanical properties of PEKK and other prosthetic materials.

Properties	PEEK	PEKK	Titanium	PMMA	Reference
Tensile strength (MPa)	100.69	115	240–890	48–62 Mpa	[24]
Elastic modulus (GPa)	3.5	5.1	103–114	3.8 × 10^3^	[24]
Flexural strength (MPa)	163.88	200	65	107–117	[3], [23], [24]
Compressive strength (MPa)	118–169	246	130–170	76 Mpa	[24]
Melting temperature (°C)	334–350	363–386	1650–1670	160	[24], [25]
Hardness	26–29 VHN	252 MPa	90 VHN	89–95 MPa	[24]
Water absorption (µg/mm^3^)	0.1–0.5	8.7	0.04	0.1–0.3	[24], [26]
Density (g/cm^3^)	1.3	FEFF1.3	4.4–4.5	1.16–1.18 g/cc	[24]

### Biological properties

PEKK shows excellent biocompatibility and has been introduced as promising alternative material for long-term orthopedic applications over titanium [10], [13], [27]. It has been approved by the FDA for oro-maxillofacial and spinal surgery [28]. In addition, PEEK is being used extensively in dentistry as a prosthetic and implant biomaterial. It offers metal-free restorations and helpful in patients with allergies [19].

As implant material, Yuan et al. [29] investigated osteointegration in PEKK in terms of chemistry and surface microstructure. It was reported that the other ketone group in PEKK increases the ability of surface chemical modification. With more ketone groups, the presence of -SO_3_H will be more on PEKK than PEEK. This leads to complex surface topography, greater surface area, and micro rough surface, which will affect the cell behavior and osteointegration on the surface of PEKK [30]. The surface modification by increasing the porosity and incorporation of HA had a positive impact on the osteointegration property [29]. Bioactive PAEK material can be achieved by modifying the surface using various bioactive ceramic such as beta-tricalcium phosphate (β-TCP), hydroxyapatite (HA), and bioactive glasses (BG). Converse et al. [31] used a combination of different methods that include compression molding, particle leaching, and powder processing to develop a HA whisker reinforced porous PEKK. In comparison to uncoated PEEK, Walsh et al. reported that coating PEEK using plasma-sprayed titanium improved the histological and mechanical properties of the bone- implant interface after implantation [32].

Regarding antibacterial activity, according to Wang [7], PEKK shows less bacterial adhesion on its surface compared to the orthopedic industry PEEK. The adherence *Staphylococcus epidermidis* were 37% less on the surface of PEKK. After five days of culture, they found around 50% decrease in the attachment and growth of *Pseudomonas aeruginosa* on PEKK compared to PEEK without using antibiotics. Also, Moore et al. [33] found the less inflammatory response from PEKK compared to PMMA in a rat study.

### Applications of PEKK in dentistry

**Fig. 2:**
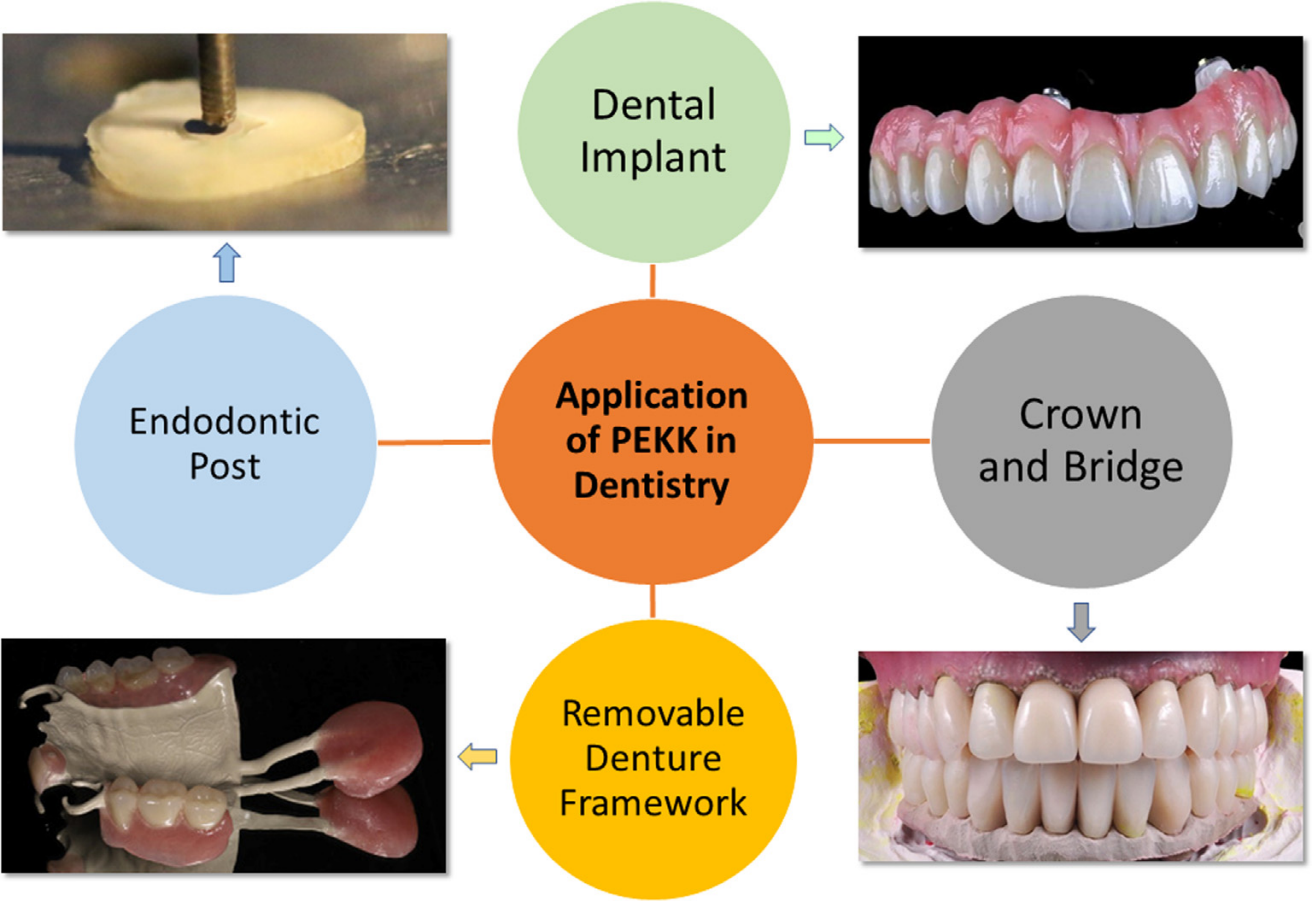
Current and potential applications of PEKK in dentistry.

The PEKK has been successfully used in dentistry as a prosthetic and implant biomaterial. Recently, PEKK has been applied in various areas of dentistry due to suitable mechanical, fracture resistance, shock-absorbing, and better stress distribution [17], [34], [35], [36], [37]. The PEKK has excellent biocompatibility as it offers metal-free restorations, and it is considered as an alternative to metal and ceramics [19]. Fig. 2 shows the various applications of PEKK in dentistry.

### PEKK as a prosthetic material

**Fig. 3:**
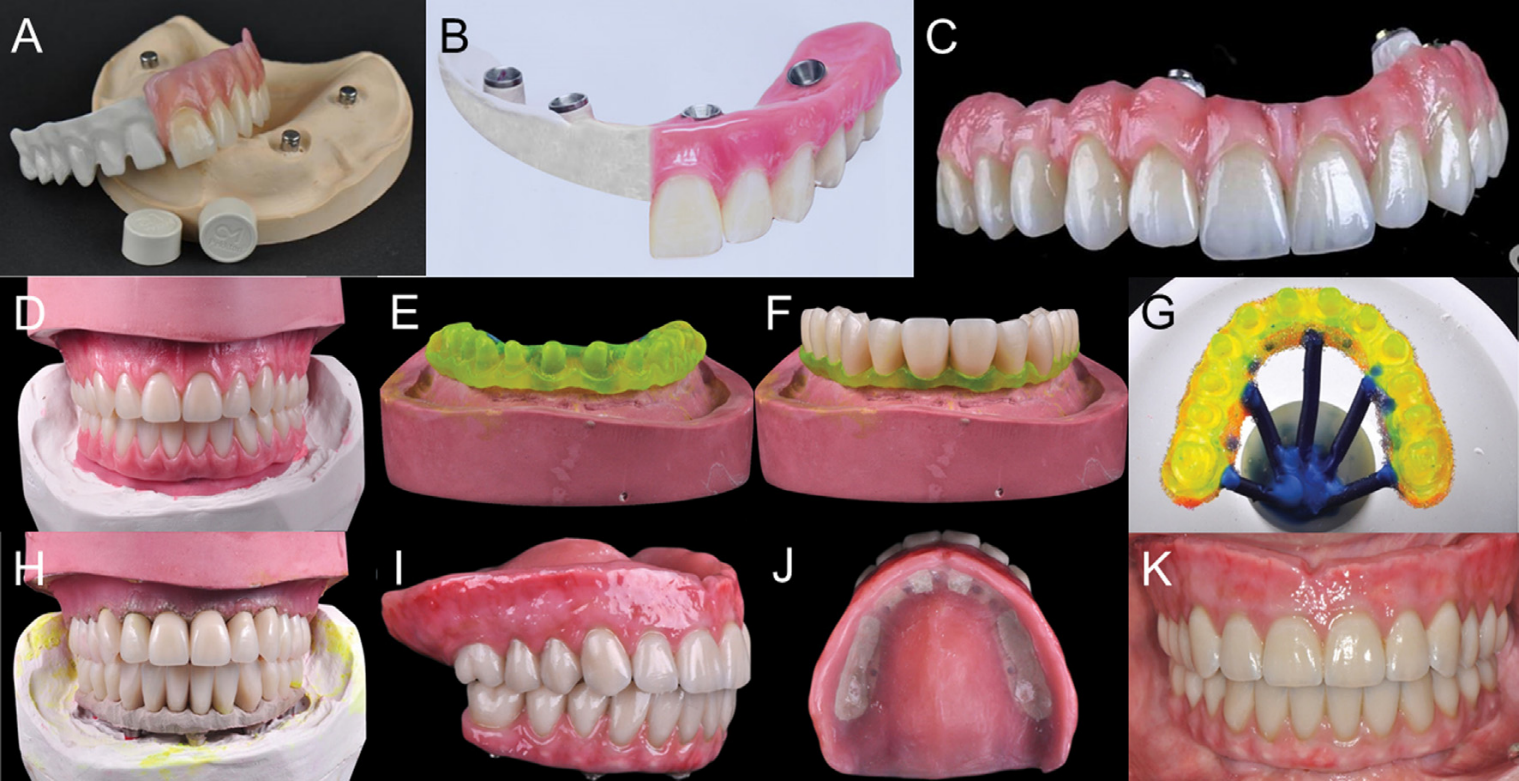
(A–C) Various PEKK implant prostheses made by Pekkton (Cendres + Métaux, Switzerland) containing monolithic and bi-layered veneered resin; (D–K) Use of PEKK for implant prosthesis framework with single lithium disilicate glass-ceramic crowns [62]. (D) Diagnostic tooth arrangement following the duplication of maxillary and mandibular interim dentures. (E) Maxillary framework resin pattern. (F) Maxillary teeth fitted on resin pattern framework. (G) Maxillary resin pattern framework for investment. (H) Teeth fitted over PEKK frameworks on maxillary and mandibular casts. (I) Maxillary complete denture, and mandibular implant-supported prosthesis. (J) Tissue surface of the maxillary denture. (K) Frontal view of the completed maxillary complete denture and mandibular implant-supported prosthesis.

The PEKK has low density, low elastic modulus, high strength, and acceptable wear resistance. It can be a potential material for application as a restorative material in fixed prosthodontics [34], [38]. Computer-aided design (CAD) and computer-aided manufacturing (CAM) technologies have increased accuracy and made it easier for the fabrication of modern restorative and prosthetic materials [39], [40], [41]. Individual ceramic produced from CAD/CAM can be incorporated in the complete denture to increase their wear resistance [40], [41], [42]. Recently, CAD/CAM technologies are used in the fabrication of PEKK prosthetic restorations [39], [43], [44]. Pekkton® ivory (PEKK) is used for monolithic and bi-layered material with an indirect composite veneer (Fig. 3A–C) [17].

Bonding of PEKK to restorative materials is essential in restorative and prosthetic dentistry. Various surface treatment methods of PEKK have been formulated for bonding using various adhesives systems [6], [45], [46], [47], [48], [49]. Lee et al. [45] studied the PEKK’s bond strength (shear) to dental resin composite by using various surface treatment methods for PEKK bonding and found that mechanical surface treatment behaves better than chemical surface treatment (95% sulfuric acid and air abrasion using alumina with 110 µm and 50 µm). Unlike other adhesives, 10-methacryloyloxydecyl dihydrogen phosphate and self-etching universal adhesive containing silane (Single Bond Universal) presented efficient shear bond strength in all treatments. Similarly, the non-thermal plasma surface modification with sandblasting increased the shear bond strength between the resin cement and PEKK [50]. Universal adhesive shows similar bonding as visio-link (light-polymerizing PMMA and composite resin primer) for PEKK [47].

The fit of the dental restoration is another important factor in the prosthetic dentistry. Poor marginal fit results in plaque deposition, recurrent caries with periodontal damage, and failure of the restoration [51], [52], [53]. Several researchers have proposed 24–110 μm as the acceptable marginal discrepancy for CAD/CAM fixed restorations [54], [55]. Bae et al. [39] studied the three-dimensional marginal fit (internal fit) of PEKK 51.64 ± 1.5 (36.12 ± 1.34) μm and zirconia copings 69.62 ± 8.11 (41.6 ± 1.63) μm and observed that the marginal fit (internal fit) were within the acceptable range. However, the PEKK presented less stress distribution around its loading areas, and better fitness was observed in the PEKK coping compared to zirconia coping [39].

Tooth/enamel wear caused by dental restorations is common and varies with the type of restorative materials. Ideally, the tooth/enamel wear due to dental restorations should not be more than the physiological wear of the teeth. The selection of appropriate restorative materials, aimed at having an almost similar degree of hardness to that of enamel is important for minimizing/retarding the harmful and irreversible consequences of tooth/enamel wear [56], [57]. Choi et al. [58] evaluated the effects of polymeric restoration on opposing tooth wear where they examined five materials fabricated from CAD/CAM: Pekkton (PEKK), Yamahachi PMMA (YAP), Mazic Duro (MZD), Vipi Block Monocolor (VBM), and Vita Enamic (ENA). It was seen that PEKK resulted in the most significant material wear but the least antagonist tooth wear. PEKK, YBM, and YAP were easily deformed and displaced by stress due to low elastic modulus. Therefore, appropriate restorative material should be selected for a specific clinical situation. Therefore, crowns fabricated from PEKK material show high wear than zirconia crowns [58], [59].

### PEKK as an implant biomaterial, abutments, and prosthesis

The high-performance with *iso*-elastic characteristics of PEKK has potential applications in oral implantology [19]. PEKK has the advantage of being enough strength, lightweight, wear resistance, and elastic modulus close to that of dentin [34]. Dental implants fabricated from thermoplastic resins have also shown acceptable results for the percentages of bone contact [60]. In oral implantology, PEKK can be used as implant abutments [27], [61], framework material for implant prosthesis [17], [62], [63], [64], prosthetic crown materials over the implant [17], and implant biomaterial [27]. PEKK is metal-free and presents an alternative material to titanium implant [27]. The advantage of PEKK abutments is adjustable and compatible with various veneering materials [17] and can be used as a framework for an implant-supported prosthesis [65]. Combining the PEKK attachment system with titanium can be a potential material to provide long-term retention in implant prosthesis [66].

**Fig. 4:**
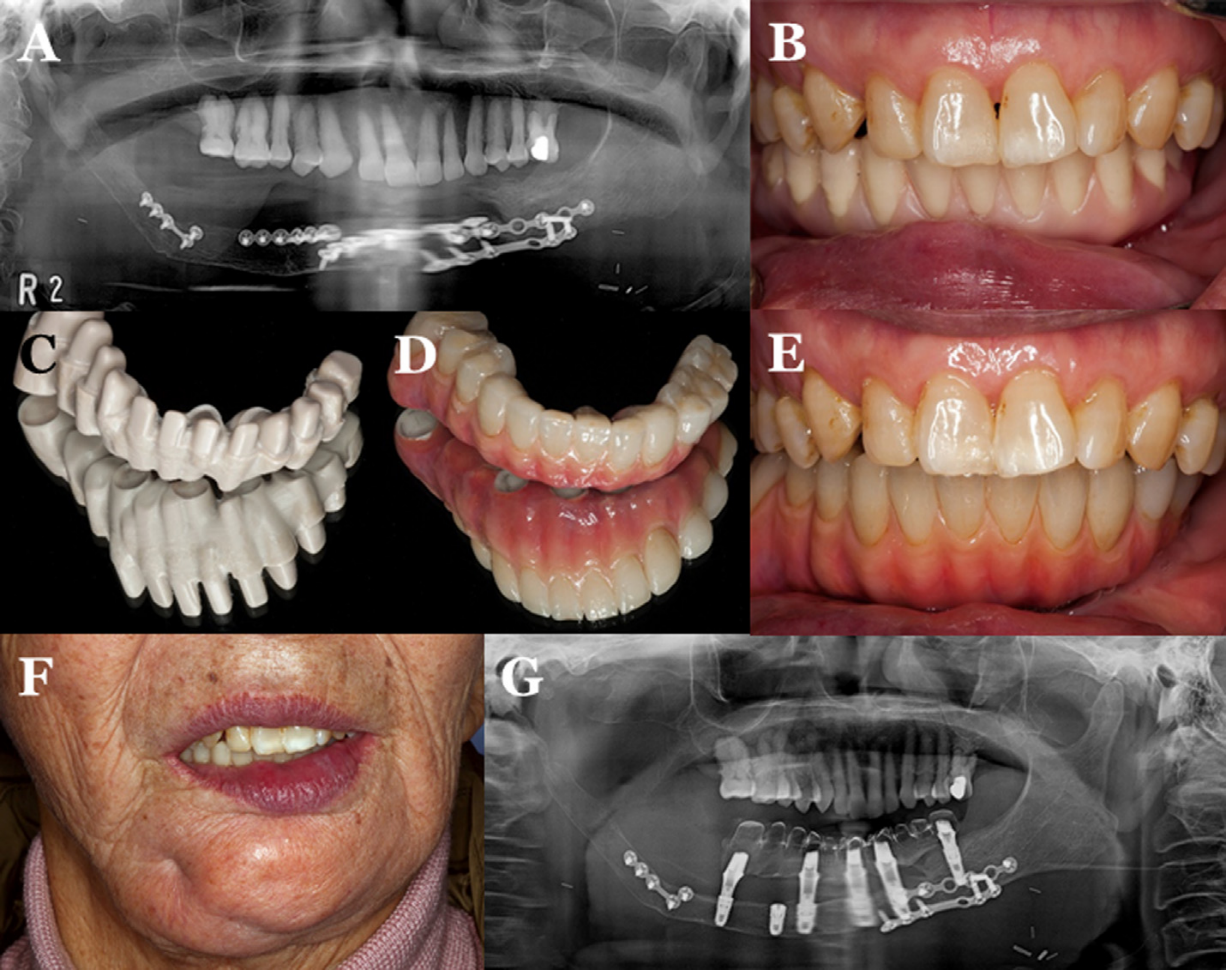
PEKK framework material for rehabilitation of a mandibulectomy patient [63]. (A) Panoramic radiograph of mandible following reconstruction with fibula free flap. (B) Trial denture. (C) PEKK framework fabricated after scanning the completed wax trial denture. (D) Final prosthesis after adding gingiva opaquer and gingiva-colored resins to match the gingiva, and cementing PMMA crowns to the framework. (E) Intraoral view of the prosthesis. (F) Final prosthesis in the patient. (G) Panoramic radiograph at follow up visit.

Conventional complete denture (CCD) opposed by an implant-supported fixed complete dental prosthesis (ICFDP) presents the problem of replacement of the posterior teeth due to wear of acrylic teeth in a few years following insertion. This problem is seen more in complete denture (47.7%) followed by ICFDP (19.6%) [67], [68], [69]. To overcome these problems, CAD/CAM zirconia teeth can be incorporated in a complete denture or another implant prosthesis to increase their wear resistance [40], [41], [42], [70], [71]. Dawson et al. [62] described the application of PEKK as a framework for ICFDP with single lithium disilicate glass-ceramic crowns and a CCD (Fig. 3D-K). The prosthesis provides a non-CAD-CAM option for the fabrication of ICFDPs and CCD with single ceramic crowns. PEKK can also be used in maxillofacial rehabilitation. Oh et al. [63] described the rehabilitation of a mandibulectomy patient with fibula free flap and implant-supported prosthesis using PEKK framework material (Fig. 4).

The PEKK framework shows less stress to the implant and tissue under compressive stress compared to tensile stress [37]. Therefore, the PEKK framework should be limited in some areas are they are resilient framework. The rigid framework prosthesis shows favorable stress distribution. Although there is a wide application of PEKK in oral implantology, they should be applied for a suitable purpose, and further studies are necessary for studying the chemical modulation of PEKK to increase the implant-contact.

### PEKK for fabricating removable partial frameworks and attachments

**Fig. 5:**
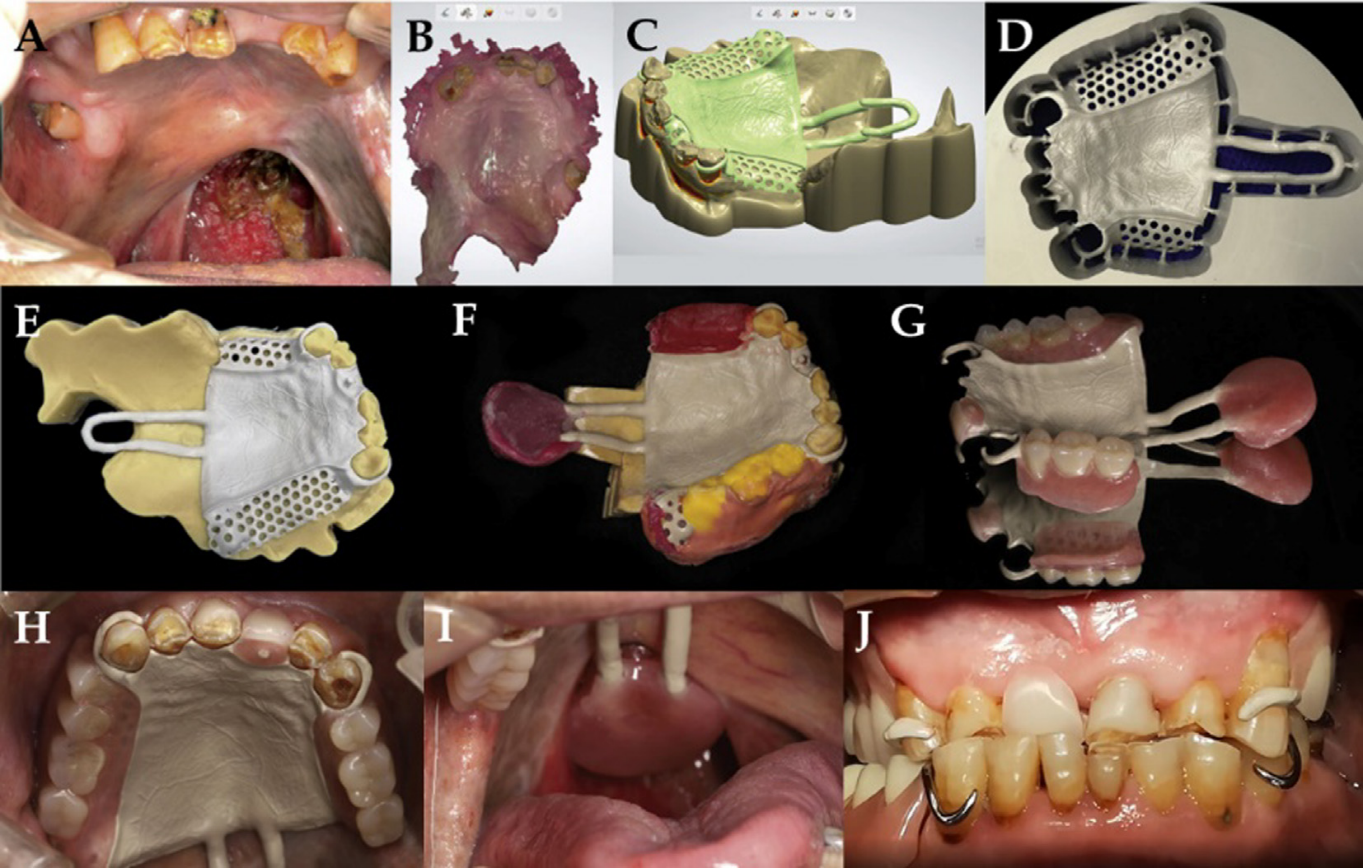
Digital workflow for the fabrication of a PEKK in removal speech bulb prosthesis [38]. (A) Pretreatment maxillary arch with a defect of the soft palate. (B) Intraoral scan of the maxillary arch. (C) Computer-aided design of PEKK framework with showing components design of PEKK framework. (D) Computed-aided manufacturing showing milled framework in PEKK disk. (E) PEKK framework on the master cast. (F) Maxillary edentulous area definitive impression, maxillo-mandibular record, and impression of the soft-palate defect. (G), Final PEKK removable prosthesis. (H) Maxillary major connector. (I) PEEK rod and acrylic bulb of the prosthesis. (J) Intraoral view of the removable prosthesis with speech bulb.

Metal clasps in removable prosthesis carry disadvantages of being unaesthetic and may cause oral galvanism and allergic reactions in some patients [72]. Thermoplastic materials have solved such problems to some extent [73], [74]. Recently, PEKK is used in removable partial denture (RPD) as dental clasps and frameworks using digital technology. Sun et al. [38] presented a digital workflow for applying PEKK in removal speech bulb prosthesis. The process consisted of intraoral scanning, 3D printing, designing, manufacture (digital milling of PEKK framework), and delivery (Fig. 5).

Retention is important in RPD prosthesis, Tannous et al. [75] studied the retentive force of CoCr alloy and three thermoplastics; PEEK, PEKK, and polyoxymethylene (POM). They fabricated 1.0 mm thick CoCr clasps in 0.25 mm undercuts and 1.0 or 1.5 mm thick thermoplastic resins in 0.5 mm undercuts, respectively. They found that all clasps showed high retentive force in the first period of cycling with a decrease till the end of the cycling. The resin clasps showed significantly lower retentive force than the CoCr clasps. Thermoplastic resin clasps-maintained longer retention with less retention than CoCr clasp. PEKK clasps can be used to provide retention for a longer duration. PEKK can also be used as inserts in the removable partial denture. Choi et al. [76] studied the attachment systems with a PEKK insert and found that PEKK insert showed less retention change and abrasion compared to the nylon inserts.

A finite element study by Keilig et al. revealed that there was a great influence of stress evenly distributed in the framework material of small bridges (three and four units). Furthermore, the surrounding tissues were not influenced by strain around them due to the choice of the material. This confirmed that the polymer PEKK could be an alternative to metal framework [77].

### PEKK for endodontic posts-core and endocrowns

**Fig. 6:**
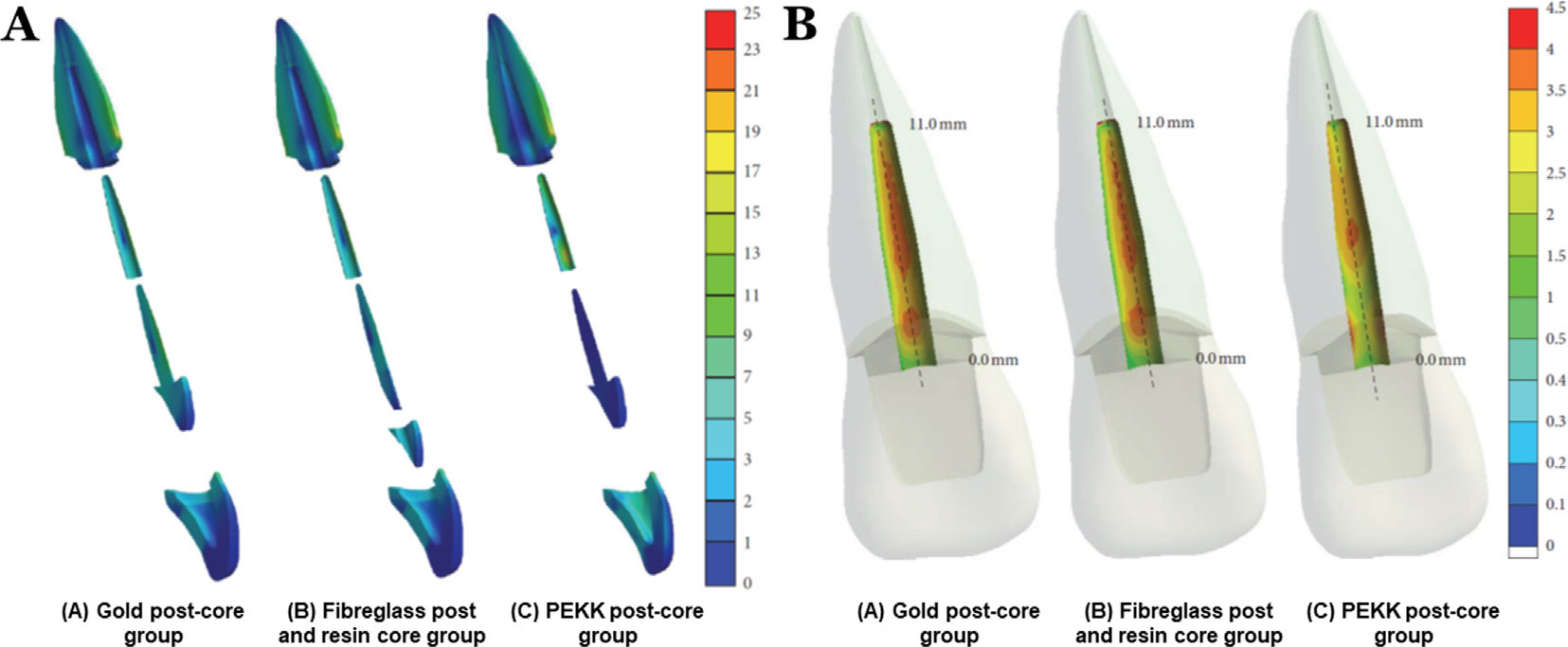
Stress distribution of the PEEK post compared to gold post-core and fiberglass post with resin core [78]. (A) Sagittal section views of stress distribution of the components. (B) Coronal section views of the stress distribution at the labial side interface surface of dentine and post cement along the mid-plane.

The PEKK biomaterial has been attracted in the post-core systems because of its acceptable processing (milling and pressing), suitable mechanical strength, and shock-absorbing ability [78], [79]. PEKK presents superior biomechanical behavior compared to metal and fiberglass post-core systems. The PEKK showed superior fracture resistance compared to metal and fiberglass post-core systems due to lower elastic modulus and flexural strength. Lee et al. [78] studied long term safety and biomechanical behavior of PEKK as intraradicular post and core material. Their study concluded PEKK as a dental post-core system has potentially high fracture resistance, although PEKK has a significantly lower elastic modulus and flexural strength than metal (gold) and fiberglass (Fig. 6). Thus, the PEKK post-core exhibited a favorable stress distribution at the intraradicular surface, indicating a less chance of root fracture than for conventional post-core materials**.** In addition, PEKK transferred higher stresses to the interface materials. Therefore, the probability of crown and cement debonding failure would be at interface level than that of rigid post-core systems where fracture of root might be anticipated [78]. PEKK post-core presents favorable stress distribution, reducing the possibility of root fracture. Nevertheless, debonding and crown failure may be higher in PEKK post-core due to its flexibility. PEKK material act as a stress breaker and reduces the forces transferred to the restoration and tooth-root [15]. Hence, PEKK can be used as endocrowns for endodontically treated teeth. This is important, especially in extensively damaged teeth.

In addition, PEKK posts are suitable for posts fabrication. Güven et al. [79] studied on the bonding of prefabricated PEKK posts, and custom made PEKK posts and conventional fiber posts. They found that the custom fabricated PEKK posts showed higher bond strength than prefabricated PEKK posts. The custom made PEKK post showed the maximum bond-strength (17.34 MPa) in the cervical region as confirmed from the scanning electron image. The conventional fiber posts showed the highest bond-strength values in the middle (11.53 MPa) and apical sections (6.86 MPa).

The conditioning of a material influences the bonding of PEKK. Fuhrmann et al. [15] evaluated the bond strength of adhesive systems to amorphous and crystalline PEKK and fiber-reinforced PEEK using five types of surface conditioning techniques. They found that the fiber-reinforced PEEK showed more considerable bond strengths and at all three storage times (5, 30, 150 days) than crystalline and amorphous PEKK. Silica coating conditioning and priming showed the highest tensile bond strength. Finally, although there is a wide application of PEKK in prosthodontics and oral implantology, long-term observation is needed as long-term data for the PEKK framework are yet not available.

## Conclusions

The PEKK materials present suitable physical, mechanical, and chemical properties and can be used for various applications such as restorative material, crown and bridge work, endo crowns, framework material for an implant-supported fixed prosthesis, and as dental biomaterial implants. Further, modifications and improving material properties can result in wider applications in clinical dentistry. Long term evaluations are needed as PEKK is recently applied in dentistry, and there are limited studies available.

## Compliance with Ethics Requirements

*This article does not contain any studies with human or animal subjects*.

## Declaration of Competing Interest

*The authors declare that they have no known competing financial interests or personal relationships that could have appeared to influence the work reported in this paper*.
